# Delta Like-1 Gene Mutation: A Novel Cause of Congenital Vertebral Malformation

**DOI:** 10.3389/fgene.2019.00534

**Published:** 2019-06-05

**Authors:** Tlili Barhoumi, Marwan Nashabat, Bandar Alghanem, AlShaimaa Alhallaj, Mohamed Boudjelal, Muhammad Umair, Saud Alarifi, Ahmed Alfares, Saad A. Al Mohrij, Majid Alfadhel

**Affiliations:** ^1^King Abdullah International Medical Research Centre, King Saud bin Abdulaziz University for Health Sciences, King Abdulaziz Medical City, Ministry of National Guard Health Affairs, Riyadh, Saudi Arabia; ^2^Medical Research Core Facility and Platforms, King Abdullah International Medical Research Center, King Saud bin Abdulaziz University for Health Sciences, King Abdulaziz Medical City, Ministry of National Guard Health Affairs, Riyadh, Saudi Arabia; ^3^Division of Genetics, Department of Pediatrics, King Abdullah International Medical Research Center, King Saud bin Abdulaziz University for Health Sciences, King Abdulaziz Medical City, Ministry of National Guard Health Affairs, Riyadh, Saudi Arabia; ^4^Medical Genomics Research Department, King Abdullah International Medical Research Center, King Saud bin Abdulaziz University for Health Sciences, Ministry of National Guard Health Affairs, Riyadh, Saudi Arabia; ^5^Department of Zoology, College of Science, King Saud University, Riyadh, Saudi Arabia; ^6^Department of Pediatrics, College of Medicine, Qassim University, Buraidah, Saudi Arabia; ^7^Department of Surgery, College of Medicine, King Saud bin Abdulaziz University for Health Sciences, Ministry of National Guard Health Affairs, Riyadh, Saudi Arabia

**Keywords:** bone development, congenital vertebral malformation, Delta Like-1, Notch signaling pathway, scoliosis

## Abstract

Skeletal development throughout the embryonic and postnatal phases is a dynamic process, based on bone remodeling and the balance between the activities of osteoclasts and osteoblasts modulating skeletal homeostasis. The Notch signaling pathway is a regulator of several developmental processes, and plays a crucial role in the development of the human skeleton by regulating the proliferation and differentiation of skeletal cells. The Delta Like-1 (DLL1) gene plays an important role in Notch signaling. We propose that an identified alteration in DLL1 protein may affect the downstream signaling. In this article, we present for the first time two siblings with a mutation in the DLL1 gene, presenting with congenital vertebral malformation. Using variable *in silico* prediction tools, it was predicted that the variant was responsible for the development of disease. Quantitative reverse-transcription polymerase chain reaction (PCR) for the Notch signaling pathway, using samples obtained from patients, showed a significant alteration in the expression of various related genes. Specifically, the expression of neurogenic locus notch homolog protein 1, SNW domain-containing protein 1, disintegrin, and metalloproteinase domain-containing proteins 10 and 17, was upregulated. In contrast, the expression of HEY1, HEY2, adenosine deaminase (ADA), and mastermind-like-1 (MAML-1) was downregulated. Furthermore, in a phosphokinase array, four kinases were significantly changed in patients, namely, p27, JANK1/2/3, mitogen- and stress-activated protein kinases 1 and 2, and focal adhesion kinase. Our results suggest an implication of a DLL1 defect related to the Notch signaling pathway, at least in part, in the morphologic abnormality observed in these patients. A limitation of our study was the low number of patients and samples. Further studies in this area are warranted to decipher the link between a DLL1 defect and skeletal abnormality.

## Introduction

During embryonic development, mesenchymal precursor bone cells determine the skeletal structure and characteristics in terms of shape, size, orientation, and integration. Human bone tissue is a dynamic structure that is continuously remodeled and balanced between degradation and renewal. This process is modulated by two major types of bone cells, namely, osteoblasts and osteoclasts of mesenchymal and hematopoietic origin, respectively (Panaroni et al., 2014). Desynchronization of the action of osteoblasts and osteoclasts leads to bone malformation. Extracellular and intracellular signals are required for the differentiation of mesenchymal cells to contribute to the osteoblastic lineage (Canalis et al., 2003). The osteoblast differentiation and bone modeling pathway are complex. Most of the studies in the literature describing the pathway depended mainly on cell line models, which resulted in significant limitations. Although not well described, numerous proteins contribute to this process to assure the integrity of the developing bone. One of the critical regulators of tissue renewal and embryonic development is the Notch signaling pathway, which mediates cell-to-cell interactions in this process (Zanotti and Canalis, 2012). However, the definite mechanisms involved in this diverse and complex role of Notch remain to be clarified (Muguruma et al., 2017).

**FIGURE 1:**
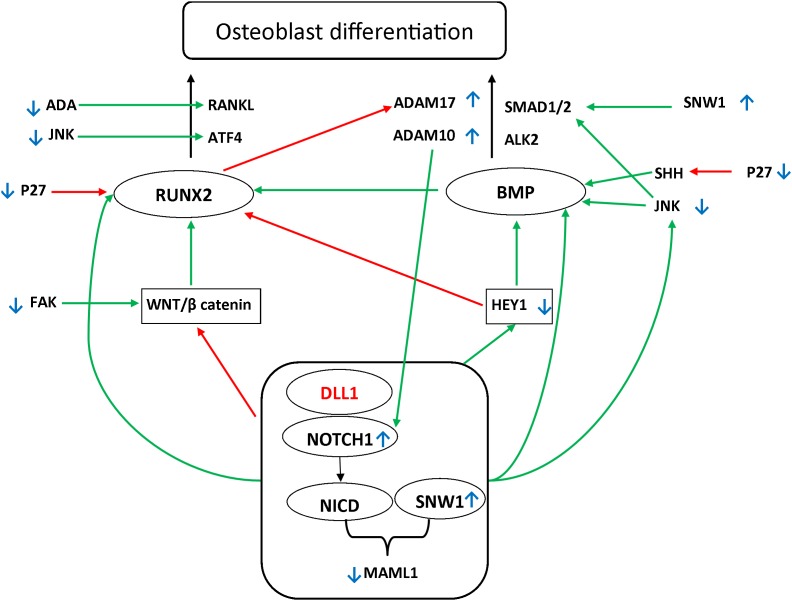
Possible pathways underlying the DLL1/Notch signaling pathway defect. Two main pathways have been shown to be involved in osteoblast differentiation: Runt-Related Transcription Factor 2 (RUNX2) (OMIM 600211)/Receptor Activator of NF-kappa-β Ligand (RANKL) (OMIM 602642) and TGFβ Bone morphogenetic protein (BMP). Mothers against decapentaplegic homolog (SMAD) further activates the RUNX2. BMP2 (OMIM 112261) and BMP9 (OMIM 605120) are major regulatory factors in (Date et al., 2004; Sharff et al., 2009) modulating the osteogenic differentiation of bone mesenchymal stem cells, advancing mineralization, increasing adherence, and facilitating the activation and expression of several markers associated with osteogenesis (Sun et al., 2015). Among the main regulators affecting these pathways are WNT/β-catenin and HEY1/2 (OMIM 602953 and 604674, respectively). It has been reported that RUNX2 is upregulated by the WNT/β-catenin pathway. Moreover, it has been shown that RUNX2 is downregulated by the Notch signaling pathway. Alternatively, Notch upregulates hairy-and-enhancer-of-split-1–7 (HES1–7) and HEY1/2. These play a regulatory role downstream in the process by activating BMP, in addition to the negative feedback on RUNX2 (Sharff et al., 2009; Zeng et al., 2015; Wang et al., 2016). The Notch signaling pathway further orchestrates the process of osteoblast differentiation through direct upregulation of RUNX2, BMP, and JNK, which activate the SMAD1 receptor by phosphorylating the linker region (Swiatek et al., 1994; Zamurovic et al., 2004; Canalis et al., 2014; Li et al., 2015; Cao et al., 2017; Kibe et al., 2018). Green arrows indicate the upregulation of the targets. Red arrows indicate the downregulation of the targets. Small blue, up and down arrows reflect the results in the affected siblings.

The Notch signaling pathway is a particularly conserved pathway that regulates cell fate determination and skeletal development. It is activated following proteolytic cleavage of the Notch receptor, after interactions between Notch and the correspondent ligand, which induce the release of the Notch intracellular domain to the nucleus (Watanabe et al., 2003). Canonical Notch ligands can be divided into the following: Jagged-1, Jagged-2, Delta Like-1 (DLL1) [Online Mendelian Inheritance in Man (OMIM) 606582], Delta Like-3 (DLL3) (OMIM 602768), and Delta Like-4 (DLL4) (OMIM 605185) (Zanotti and Canalis, 2010, 2013; Sukarawan et al., 2016). After cleavage, the intracellular domain multimerizes with SNW domain-containing protein 1 (SNW1), and promotes the expression of several target genes. This is achieved by controlling a transcriptional activation complex that includes the specific coactivator Mastermind Like-1 (MAML-1) (OMIM 605424) (Nam et al., 2006; Wilson and Kovall, 2006; Vasquez-Del Carpio et al., 2011; Watanabe et al., 2013). Activation of Notch signaling through DLL1 interaction induces cell differentiation and proliferation, which regulate cell patterning, lineage, specification, and morphogenesis (Jaleco et al., 2001; Muguruma et al., 2017). Figure 1 summarizes the osteoblastic differentiation pathways related to the DLL1/Notch signaling pathway.

Genes of the Notch signaling pathway are implicated in numerous diseases presenting with skeletal anomalies. For example, mutations in the JAG1 and NOTCH2 genes were found to be responsible for the development of Alagille syndrome. Spondylocostal dysostosis (SCD) is caused by the DLL3, MESP2, LFNG, and hairy-and-enhancer-of-split-7 (HES7) genes (Giampietro et al., 2013), which are also related to the Notch signaling pathway.

Recently, it was demonstrated that the nephroblastoma overexpressed gene (OMIM 164958) and DLL1 co-regulate osteogenic differentiation in a Hey1-dependent manner through upregulation of the bone morphogenetic protein-9 (Su et al., 2018). Adenosine deaminase (ADA) (OMIM 608958) is an enzyme involved in several disorders related to immunodeficiency and skeletal malformations (Cederbaum et al., 1976; Sauer et al., 2009). ADA-deficient mice models exhibited impairment of the receptor activator of NF-kappa-β ligand (RANKL)/osteoprotegerin axis (Sauer et al., 2009).

Next-generation sequencing is becoming a useful tool for the diagnosis of rare monogenic diseases. The importance of next-generation sequencing is emphasized in consanguineous populations, in whom the prevalence of autosomal recessive diseases increases with time. This tool has been widely utilized in numerous countries to describe novel diseases and manage challenging cases (Alfares et al., 2017).

In this study, we describe a novel disease caused by a novel mutation in the DLL1 gene in two Saudi siblings. The objective of the study was to define the gene profile of DLL1/Notch signaling pathway in these patients and interaction with target genes implicated in bone formation and skeletal development, which may explain the observed skeletal malformation.

## Materials and Methods

### Patients and Study Approval

Two Saudi siblings were followed up at the genetics clinic of King Abdulaziz Medical City in Riyadh, Saudi Arabia. The study was approved by the Institutional Review Board of King Abdullah International Medical Research Center (RC18-017.R). The father of the patients signed the informed consent form approved by the Institutional Review Board.

### Whole Exome Sequencing

Whole exome sequencing was performed in an accredited commercial lab (Centogene-Germany) as follows. Approximately 37 Mb (214,405 exons) of the Consensus Coding Sequences were enriched from fragmented genomic DNA by >340,000 probes designed against the human genome (Nextera^®^Rapid Capture Exome, Illumina), and the generated library was sequenced on an Illumina platform to an average coverage depth of 100–130×. End-to-end in-house bioinformatics pipelines, including base calling, primary filtering of low-quality reads and probable artifacts, and annotation variants were applied. All disease-causing variants reported in HGMD^®^, in ClinVar, or in CentoMD^®^, in addition to all variants with minor allele frequency (MAF) < 1% in the ExAc database were considered. Evaluation was focused on coding exons along with flanking ±20 intronic bases.

### *In silico* Prediction

*In silico* prediction tools used to analyze the identified variant included SIFT^1^, Polyphen-2^2^, MutPred^3^, Provean^4^, and MutationTaster^5^.

### Peripheral Blood Mononuclear Cells (PBMCs) Isolation

Peripheral blood samples were collected from the two affected and two healthy siblings. After collection in ethylenediaminetetraacetic acid tubes, the peripheral blood mononuclear cells (PBMCs) were immediately isolated as previously described (Nilsson et al., 2008). Briefly, using Ficoll Histopaque (BD Biosciences), samples were centrifuged at 100 ×*g* for 30 min at 4°C. The upper layer of the plasma fraction was collected and immediately frozen for further processing. PBMCs, formed in the interphase between the Histopaque and medium, were aspirated and immediately processed for further investigation.

### Quantitative Reverse-Transcription Polymerase Chain Reaction (PCR) Analysis of the Notch Signaling Pathway

The RT2 Profiler polymerase chain reaction (PCR) array was used to analyze the expression of genes involved in the Notch signaling pathway. The expression of hypoxanthine phosphoribosyltransferase 1, glyceraldehyde-3-phosphate dehy drogenase, and β-2-microglobulin was used as control. For amplification, 1 μl of cDNA was used as the input with a final volume of 10 μl per reaction, and all reactions were performed in an optical 384-well plate (Qiagen). The Fast Real-time PCR system 7900HT (Thermo Fisher Scientific) was used to perform the amplification. The PCR cycling parameters were as follows: 95°C for 10 min, followed by 40 cycles of PCR reactions at 95°C for 15 s, and 60°C for 1 min. The comparative threshold cycle Ct (ΔΔCt) method was used to calculate the relative expression levels of the genes assayed in the cell synchronization validation method experiment. The Ct value of each gene was normalized to the corresponding average Ct value of the housekeeping genes for the same sample, to obtain the relative threshold cycle (ΔCt). For each biological replicate, the relative threshold cycle was exponentially transformed into ΔCt expression by calculating two raised to the ΔCt. We used the control samples for each gene ΔΔCt to calculate the average and standard deviation of the biological replicates. Finally, from the control samples, the ΔΔCt was calibrated and expressed as a relative fold-change. For the relative mRNA analysis, raw Ct values were exported from ABI 7900 and imported into Microsoft Excel. Using historical/generic values from standard curves (intersection at 0 = 40, slope = -3.5) (data not shown), Ct values were used to calculate the copy number for each well. The copy numbers were subsequently normalized to the average of the housekeeping genes, and the mRNA levels were expressed relative to the control samples.

### Phospho-Kinase Antibody Array

For the determination of kinase phosphorylation patterns in PBMCs protein extracts, a phospho-antibody array analysis was performed using a Proteome Profiler Human Phospho-Kinase Array Kit (ARY003B; R&D Systems, Minneapolis, MN, United States). Briefly, PBMCs from the controls or patients were lyzed using Lysis Buffer 6 (R&D Systems, Minneapolis, MN, United States) and gently agitated at 4°C for 30 min. The samples were microcentrifuged at 14,000 ×*g* for 5 min and subsequently, the supernatant was transferred into a new tube to determine the concentration of proteins. The cell lysates were immediately used. Human Phospho-Kinase Array nitrocellulose membranes were blocked and incubated with cellular extract overnight on a shaking platform at 4°C. To remove the excess unbound proteins, the membranes were washed thrice for 10 min with 1× Wash Buffer, and incubated with detection antibody cocktails for 2 h at room temperature on a shaker. Subsequently, membranes were incubated with Streptavidin-HRP for 30 min on a shaker. Three additional washes (10 min per wash) were performed, and the membranes were incubated with chemiluminescent detection reagents to detect spot densities. The Chemi Reagent Mix was added for 1 min, and spots were visualized using a Biospectrum Imaging System. The ImageJ software was used to perform the densitometry analysis and visualization of proteins.

### Protein Extraction and Western Blotting

Protein extraction and western blotting were performed as previously described (Tamgue et al., 2019). Skin fibroblasts collected from healthy donors or patients were cultured in RPMI 1640 medium supplemented with 10% serum, 10% fetal bovine serum, antibiotic, 20 pg/ml of fibroblast growth factor, and 1% insulin. Cells were lyzed with lysis buffer (RIPA buffer; Sigma) and subjected to electrophoresis in 10% polyacrylamide gradient gels. Western blotting analysis was performed with antibodies against anti-DLL1 (MAB1881). All antibodies were purchased from Cell Signaling. Signals were detected using a ChemiDoc MP System (Bio-Rad) and the ImageLab software. Sample loading was assessed by probing the same membrane with anti-glyceraldehyde-3-phosphate dehydrogenase antibody (Cell Signaling).

### Immunofluorescence

Immunofluorescence microscopy was performed as previously described with modifications (Mian et al., 2016).

### Flow Cytometric Analysis

The flow cytometric analysis was determined as previously described (Barhoumi et al., 2017; Caillon et al., 2017) with modifications.

## Results

### Clinical Description of the Cases

#### Patient 1 (Figure 2A,B)

**FIGURE 2:**
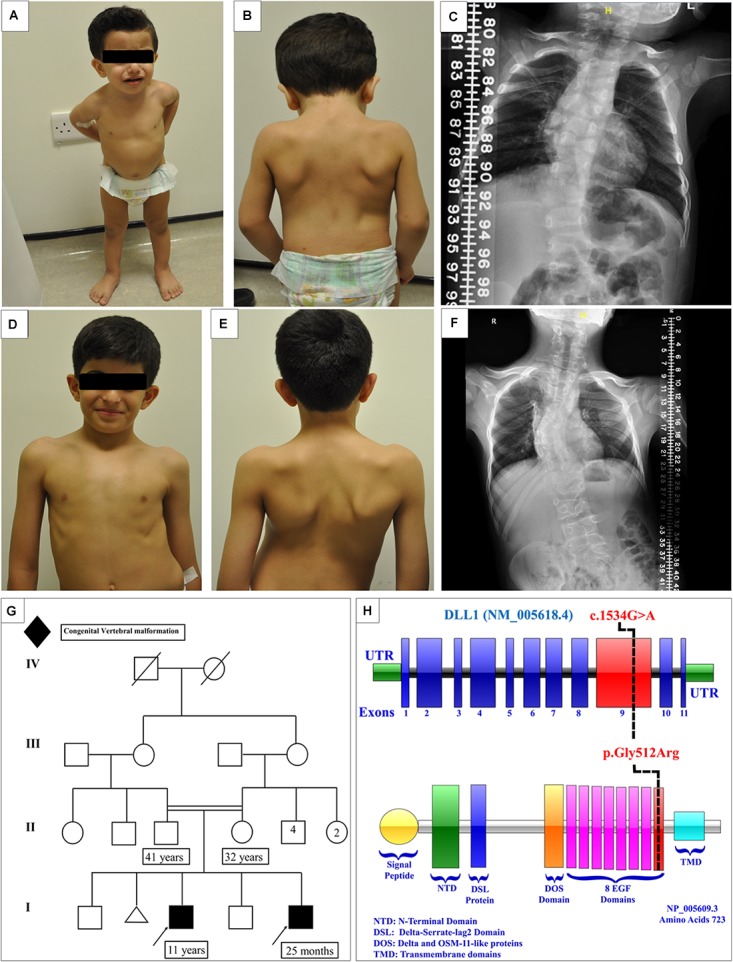
Clinical features of the affected siblings. **(A,B)** Clinical features of patient 1. **(C)** The latest scoliosis survey for patient 1. **(D,E)** Clinical features of patient 2. **(F)** The latest scoliosis survey for patient 2. **(G)** Family pedigree. **(H)** DLL1 gene and protein showing the site of the identified variant in the epidermal growth factor-like (EGF) domain 8.

A male delivered through Cesarean section (C/S) due to history of previous C/S. The parents are first cousins (Figure 2G). The antenatal follow-up was unremarkable. A detailed antenatal ultrasound was performed late in the third trimester, which limited the evaluation of many organs including the heart, chest, upper and lower limbs, and spine. Following delivery, the patient was discharged with the mother in good health, except for left scrotal swelling. The first concern was reported at the age of 2 months, when the parents noticed that the scrotal swelling did not improve and became irreducible. The patient was referred to pediatric surgery for evaluation. The examination revealed the presence of scoliosis and multiple anomalies in the spine. All growth parameters were around the 50th percentile. The developmental milestones were appropriate for the age of the patient.

The initial skeletal survey showed multiple vertebral body anomalies involving the thoracic spine, fused T4–T5 on the right side, and bifid T6 and T8 and fused T11–T12 on the left side. In addition, mild dextrocurve convexity involving the thoracolumbar spine was observed. The Cobb angle from the superior endplate of T12 to the inferior endplate of L1 was 21°. There were fused ribs at the level of the right third and fourth ribs. The patient’s elder brother exhibited identical spinal anomalies, which raised the suspicion of a familial genetic disorder. The proband and his affected brother were referred to genetic service at the age of 4 months and 10 years, respectively, to evaluate the underlying cause of their vertebral spine anomalies.

Currently, the patient is 25 months old. He was admitted to the hospital several times due to a recurrent chest infection. He exhibits normal developmental milestones. The current growth parameters are as follows: height, 84 cm (25th–50th percentile); weight, 11.4 kg (50th percentile); and head circumference, 50 cm (50th percentile). Examination showed that the patient is vitally stable without facial dysmorphic features. The patient has apparent scoliosis. The remaining systemic examination and all biochemical investigations were unremarkable. The follow-up scoliosis series X-rays did not show progression of the disease (Figure 2C). Whole exome sequencing showed a homozygous missense variant in the DLL1 gene c.1534G > A (p.Gly512Arg) (Figure 2H) in the proband and his affected brother. Segregation analysis was performed for the parents, who were heterozygous carriers, and for other unaffected family members. The results showed that none of them were homozygous carriers of the variant.

#### Patient 2 (Figure 2D,E)

A male delivered through C/S due to previous C/S. Examination during the antenatal period was unremarkable. At the age of 1 month, multiple congenital anomalies were detected in the spine. The first spine X-ray showed multiple vertebral congenital abnormalities in the segmentation of the dorsal and lumbar vertebrae, in the form of a butterfly hemivertebra, and mainly involving the dorsal spine and upper lumbar spine. All the developmental milestones were appropriate for the age of the patient. The growth parameters were around the 50th percentile.

**FIGURE 3:**
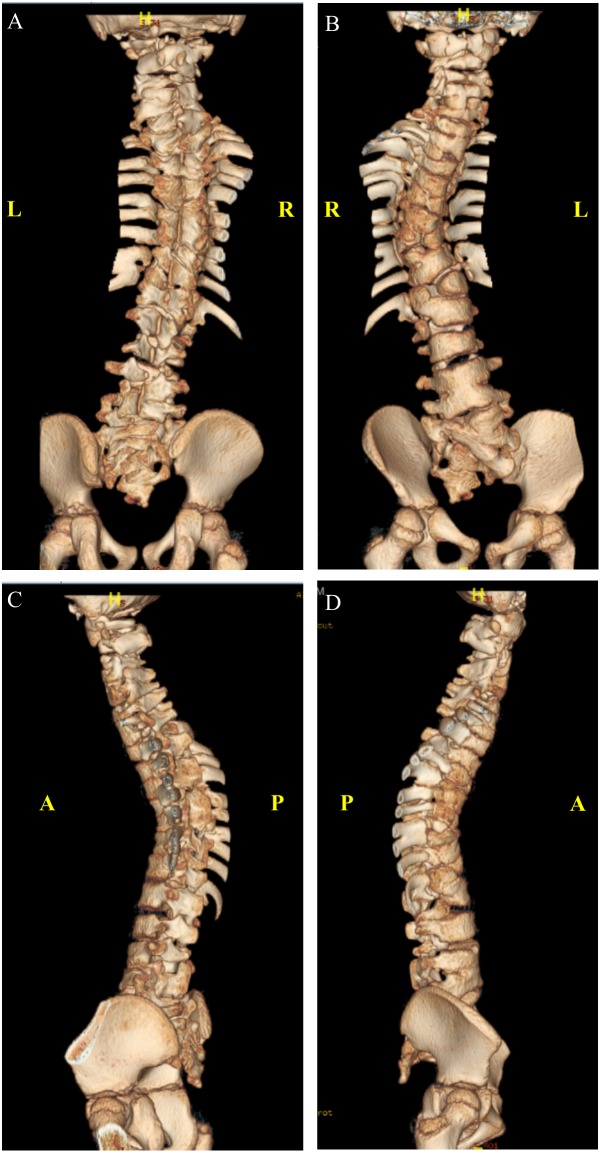
Three-dimensional computed tomography (CT) scan. **(A)** Anterior. **(B)** Posterior. **(C)** Left lateral. **(D)** Right lateral view.

At the age of 4 years, whole spine magnetic resonance imaging in addition to spine computed tomography (Figure 3) was performed, showing S-shaped scoliosis of the thoracolumbar spine with kyphotic deformity centered at T5. Multiple segmental fusion anomalies of the cervical, thoracic, and lumbar spines including the hemivertebra and block vertebra, as well as a fusion of the posterior elements (especially at the C4–C5, C6–C7, T3–T4, and T5–T6 levels), bifid odontoid process, and fusion of midribs on both sides were observed.

The condition of the patient is stable, with a static progression of scoliosis (Figure 2F). As mentioned earlier in this article, the patient is a homozygous carrier of the mutation *DLL1* c.1534G > A (p.Gly512Arg).

### Whole Exome Sequencing and *in silico* Studies

Whole exome sequencing showed a homozygous variant in the DLL1 gene c.1534G > A (p.Gly512Arg). An additional filtration process based on the family pedigree was applied to search for shared homozygous variants in the affected siblings, heterozygous variants in the parents, and either absent or heterozygous variants in the non-affected siblings. The analysis identified, in addition to the DLL1 variant, 11 variants: six intronic variants and five variants upstream or downstream the gene (Supplementary Table S1).

The results of the *in silico* prediction tools were as follows: MutationTaster predicted the variant to be disease-causing. SIFT predicted tolerance (score: 0.08), Polyphen-2 predicted probable damage (score: 1.00), and MutPred2 predicted high pathogenicity (score: 0.842).

### Identification of Differentially Expressed Genes in PBMCs From Patients With the DLL1 Gene Defect or Controls (Figure 4)

We performed a microarray analysis and detected the differential expression of 70 genes in PBMCs obtained from patients with the DLL1 gene defect versus controls. The identified genes were related to the Notch signaling pathway. The most significantly downregulated genes in patients with the DLL1 gene defect versus controls were HEY1, ADA, and MAML1. Notably, the expression of HEY2, SMO (OMIM^®^601500), and PSENEN was not significantly decreased. In contrast, the expression of four genes, namely, neurogenic locus notch homolog protein 1 (NOTCH1) (OMIM^®^190198), SNW1 (OMIM^®^603055), disintegrin and metalloproteinase domain-containing protein 10 (ADAM10) (OMIM^®^602192), and ADAM17 (OMIM^®^603639) was increased; however, the difference was significant only for NOTCH1 and SNW1.

**FIGURE 4:**
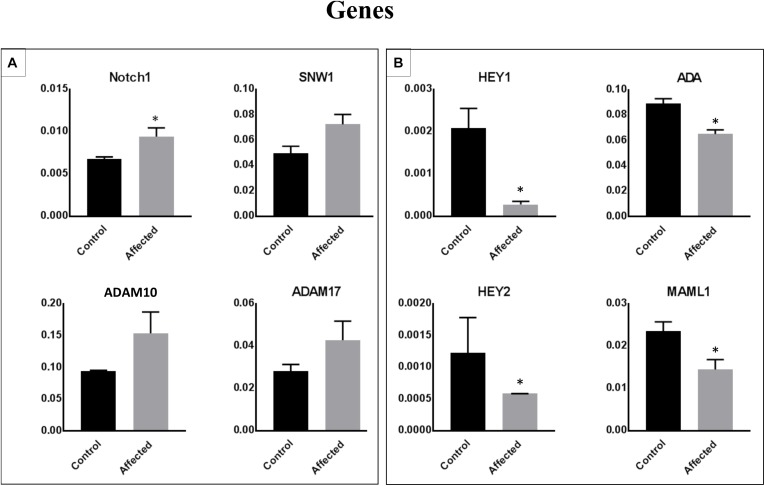
Identification of differentially expressed genes in PBMCs obtained from patients with the DLL1 defect or controls. Cell pellets harvested from PBMCs of patients or controls were used for RNA extraction and the subsequent qRT-PCR analysis. Upregulated **(A)** and downregulated **(B)** genes in patients versus controls. The ΔΔCt analysis was performed using the SDS 5.4 (Applied Biosystems). GAPDH was used as a reference control for all analyses. Data are presented as means ± SEM. Data were analyzed using the paired *t*-test. ^∗^*P* < 0.05 vs. control.

### Links of p27, JANK 1/2/3, Mitogen- and Stress-Activated Protein Kinases 1 and 2 (MSK1/2), and Focal Adhesion Kinase (FAK) to Associated Cell Signaling Pathways (Figure 5)

**FIGURE 5:**
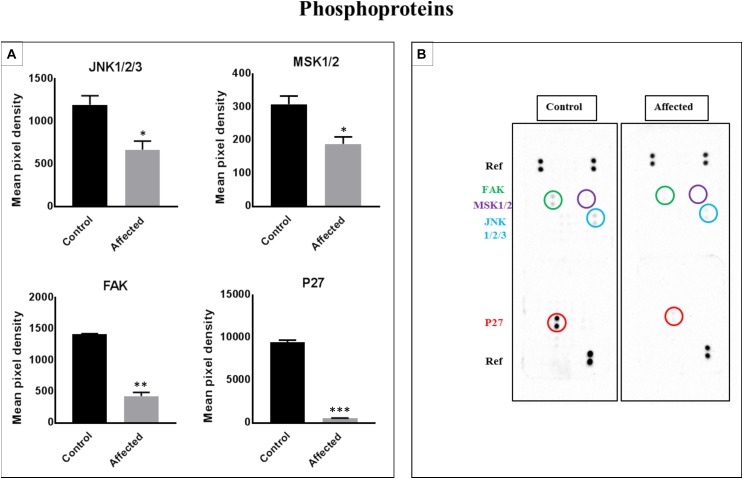
Analysis of a proteome profiler for human phospho-kinases using PBMC cell lysates obtained from patients with the DLL-1 effect and controls. **(A)** Data represent the densitometric analysis of phosphorylated signaling proteins in PBMCs. Phosphorylation levels of p27, FAK, MSK1/2, and JNK1/2/3. Data are presented as means ± SEM. Data were analyzed using the paired *t*-test. ^∗^*P* < 0.05, ^∗∗^*P* < 0.01, and ^∗∗∗^*P* < 0.0005 vs. control. **(B)** Part A and B of each array were incubated with 200 μg of cell lysate. Data shown are from a 5-min exposure.

We assessed the levels of 43 phosphorylated kinases in PBMCs obtained from patients and controls using a phosphokinase array. The purpose of this analysis was to identify candidate kinases mediating the bone defect related to the DLL1 deficiency, and determine whether the DLL1 defect may regulate the activity of phosphoproteins. Four kinases were significantly changed in patients (Figure 5A). Of note, p27 (involved in the development of growth plates and osteogenesis by modulating type I collagen, and osteoblast number and activity), Runt-Related Transcription Factor 2 (RUNX2), osteocalcin, and bone morphogenetic protein 2 (BMP2) (Yin et al., 2014) were highly phosphorylated in the controls versus the patients. The activity of c-Jun N-terminal kinase (JNK) is required for the late-stage differentiation of osteoblasts. Moreover, in general, the process of osteogenesis through BMP2 involves less phosphorylation in patients versus controls. Similar results were observed for MSK1/2 (OMIM 603607 and 603606) and FAK (OMIM 600758), which are implicated in osteoclastogenesis and bone formation, respectively (Ha et al., 2013; Yuan et al., 2018).

### Western Blotting, Flow Cytometry, and Immunofluorescence in Fibroblasts

These experiments revealed comparable localization and quantification of DLL1 between the affected patients and normal controls (Supplementary Figures S1–S4).

## Discussion

Human bone homeostasis is modulated by the Notch signaling pathway through a regulatory effect on the function and differentiation of the osteoblast and osteoclast lineages. Activation of this pathway is required during embryogenesis for adequate bone development (Zanotti and Canalis, 2016). The interaction between the DLL1 ligand and Notch receptor, as well as the downstream cascade, may affect and define the skeletal characteristics and structure. Based on the results of the present study, the Notch signaling pathway and target genes are affected in patients with the DLL1 mutation versus controls, and this disorder may explain the phenotype of these patients.

The DLL1 protein is composed of 723 amino acids, and mainly expressed on the cell membrane. The protein was described for the first time in 2003 (Clark et al., 2003). DLL1 is composed of the intracellular domain, transmembrane domain, and extracellular domain, which contains eight epidermal growth factor-like domains. The discovered mutation is located at a conserved nucleotide and amino acid number 512 (in epidermal growth factor-like 8 closer to the cell membrane) and results in the replacement of the small neutral glycine with a large, positively charged arginine. This process may affect attachment with the target protein (Figure 2H). The expression and localization of the DLL1 protein in the fibroblasts of patients were comparable to those reported for the controls.

The results showed a significant decrease in the expression of HEY1 in patients with the DLL1 defect. HEY1 regulates osteogenesis through BMP2-induced osteoblastic differentiation. Additionally, it was shown that the Notch–Hey1 pathway may inhibit osteoblastic differentiation and maturation, and that HEY1 can annul RUNX2 transcriptional activity in this process (Zamurovic et al., 2004). Despite the upregulation of NOTCH1 in the affected siblings, HEY1 was downregulated. This observation indicates that the activated Notch is the main regulator of HEY1. The downregulation of HEY1 may negatively affect the BMP pathway. However, the loss of its negative feedback effect on RUNX2 may promote the differentiation of osteoblasts. This may be helpful in patients with an impaired DLL1/Notch signaling pathway. Interestingly, in our study, we observed a decrease in the relative level of phosphorylation of JNK1/2/3 in patients versus controls, which may result in the downregulation of osteoblast differentiation via BMP2. Of note, it has been demonstrated that JNK1 is required for BMP2-induced mineralization (Liu et al., 2011). Additionally, it has been reported that inactivation of the JNK pathway does not affect the main transcription factor genes implicated in osteogenesis (i.e., RUNX2 and Osterix). However, it inhibits the mRNA of activating transcription factor 4, indicating that JNK activity is specifically required for the late-stage differentiation of osteoblasts. It is challenging to explain the decreased level of JNK phosphorylation in the affected patients, which may be attributed to an imbalance of any of the proteins involved in the pathway. A reduction in the expression of the co-activator MAML1 gene in the affected patients versus controls may be associated with an effect on the proliferation and differentiation of osteoblasts. This is achieved through stimulation of RUNX2 activity, which is essential for the maturation and proliferation of chondrocytes, and the osteogenic process. The decreased level of MAML1 may be related to the impaired DLL1 upstream. ADA is a crucial modulatory element implicated in bone cell remodeling and activity. The RANKL/osteoprotegerin axis, which is responsible for maintaining the integrity of bone, is altered in ADA-deficient animal models. Moreover, a decreased osteoclastogenesis and bone formation was related to osteoblast dysfunction (Sauer et al., 2009). The expression of the ADA gene in the current patients was significantly decreased versus the controls. The observed downregulation of ADA remains to be explained, considering that the exact role of ADA in bone development is not well established. There may be a link between the loss of negative feedback of the Notch signaling pathway on the RUNX2 and RANKL pathway and the downregulation of the ADA, which acts as a positive regulator (Whitmore and Gaspar, 2016).

As mentioned earlier, SNW1 plays an important role in osteoblast differentiation through two mechanisms: (1) by forming a preactivation complex with the Notch intracellular domain to express the downstream genes in the proliferation pathway via transcription factors such as MAML1 (Vasquez-Del Carpio et al., 2011) and (2) by activating the mothers against decapentaplegic homolog (SMAD) receptors (2 and 3) (Leong et al., 2001), through which BMPs enhance osteoblastic proliferation. The noted significant upregulation of SNW1 in the probands may be attributed to overcoming the impaired activation of Notch through the malfunctioning DLL1, and further activating the BMP–SMAD osteoblast proliferation pathway.

In addition, our results showed a marked decrease in p27 protein phosphorylation in patients versus controls. Reports have revealed that p27 is implicated in the development of growth plates, and that p27 deficiency enhances the proliferation of growth plate chondrocytes, as well as longitudinal and periosteal bone growth (Emons et al., 2006). Yin et al. (2014) reported that, in mice, p27 deficiency stimulates cell proliferation and osteoblastic bone formation. This effect is modulated by type I collagen, osteoblast number and activity, and mRNA expression of RUNX2, osteocalcin, and BMP2 (Yin et al., 2014). The proliferation of osteoblasts may also be regulated via the Sonic hedgehog signaling pathway, as demonstrated in p27-deficient mice (Liu et al., 2017). This may explain the observed downregulation of p27 in the affected siblings, possibly to counteract the negative effect of p27 and enhance bone formation. The downregulation of MSK1/2, which is involved in osteoclastogenesis via RANKL signaling and osteoclast differentiation (Ha et al., 2013), may be predicted to enhance the bone formation in patients. ADAM10 and ADAM17 are transmembrane metalloproteinases. Their expression increases significantly during BMP-induced osteoblast differentiation, which reflects the central role of ADAMs in bone development (Araya et al., 2018). Expectedly, the expression of both proteins was upregulated in the present patients, possibly to overcome the impaired Notch signaling pathway.

Additionally, the activity of FAK was also downregulated in patients. This observation was recently correlated with a decrement in bone mass in a deficient mouse model (Sun et al., 2016), in addition to osteogenic differentiation and bone formation through the WNT/β catenin pathway (Yuan et al., 2018). However, considering that the bone development pathway is complex and the functions of several elements are not fully understood, it was not possible to propose a clear explanation based on the present findings.

Muguruma et al. (2017) developed a DLL1-knockout mouse model, and noticed that the phenotype of the affected mice differed significantly from that of the wild-type mice in terms of body size. The former mice were smaller and exhibited osteosclerosis, which indicated a perturbation in the function of osteoblasts and osteoclasts. In the histopathological study, the affected mice exhibited a compromised metabolic bone turnover and a significant decrease in the osteoblast surface area and rate of bone formation (Muguruma et al., 2017). These findings are consistent with the phenotype of the present affected siblings.

Clinically, congenital vertebral malformation is considered a multifactorial disease. Although most cases are sporadic in families, the condition may be hereditary (Mackel et al., 2018). Congenital vertebral malformation can be either syndromic or non-syndromic. The non-syndromic type is considered when the spinal malformation is isolated and other body systems are spared (Turnpenny et al., 2007). Numerous genes correlated with various types of congenital scoliosis (i.e., Klippel–Feil syndrome, Alagille syndrome, SCD, spondylothoracic dysostosis, etc.) (Giampietro et al., 2013) are related to the Notch signaling pathway. The clinical manifestations of our patients and the isolated vertebral malformation, in addition to the involvement of the ribs, may relatively mimic the clinical presentation of SCD. This disorder is caused by mutations in genes related to the Notch signaling pathway, namely DLL3, MESP2, LFNG, and HES7 (Giampietro et al., 2013). Notably, patients with SCD may have short stature and experience mild respiratory distress. However, in the present patients, the heights were within the normal range and respiratory symptoms were absent. However, long-term monitoring of the respiratory function of patients is recommended. The two siblings did not exhibit any other associated symptoms, including cardiac, neurological, respiratory, genitourinary, or gastrointestinal. Additionally, they demonstrated normal intellectual abilities.

Collectively, the present results demonstrated that the Notch signaling pathway and target genes may explain, at least in part, the physiologic abnormality observed in the examined patients. This abnormality is probably established during the embryonic phase, in which the DLL1-mediated Notch downstream signaling is impaired. This disturbance leads to downregulation of the MAML1, HEY1, and ADA genes, as well as inhibition of the activity of p27, JNKs, FAK, and MSK1/2 proteins. It is suggested that these changes affect RUNX2 and the RANKL/osteoprotegerin axis, which play a crucial role in bone remodeling. A limitation of the present study was the low number of patients and samples. Therefore, further investigations in this area are warranted to explain these observations and elaborate on the link between the DLL1 gene defect and development of skeletal abnormality.

## Data Availability

The data are available from the corresponding author on request.

## Ethics Statement

This study was performed in accordance with the recommendations of King Abdullah International Medical Center Institutional Review Board. All participants provided written informed consent. The study was conducted in accordance with the tenets of the Declaration of Helsinki. The protocol was approved by the King Abdullah International Medical Center Institutional Review Board (RC18.017.R). Written informed consent was obtained from the parents of the patients for the publication of these cases.

## Author Contributions

MA, MB, and SA designed the study. TB, AlA, BA, MU, and AhA conducted the study. MN and TB collected the data. AhA, MN, and TB analyzed the data. AhA, MN, SA, and TB interpreted the data. TB and MN drafted the manuscript. MA reviewed the manuscript content. TB, MN, BA, AlA, AhA, MB, SA, MU, SA, and MA approved the final version of the manuscript. MA assumed responsibility for the integrity of the data analysis.

## Conflict of Interest Statement

The authors declare that the research was conducted in the absence of any commercial or financial relationships that could be construed as a potential conflict of interest. The reviewer AEH declared a past co-authorship with several of the authors MN and MA to the handling Editor.

## Footnotes

1 http://provean.jcvi.org/index.php

2 http://genetics.bwh.harvard.edu/pph2/

3 http://mutpred.mutdb.org/

4 http://provean.jcvi.org/index.php

5 http://www.mutationtaster.org/
