# Supplementary figures and images for: Delta Like-1 Gene Mutation: A Novel Cause of Congenital Vertebral Malformation
Source: Front Genet. 2019 Jun 5;10:534. doi: 10.3389/fgene.2019.00534 (PMC6593294; doi:10.3389/fgene.2019.00534)

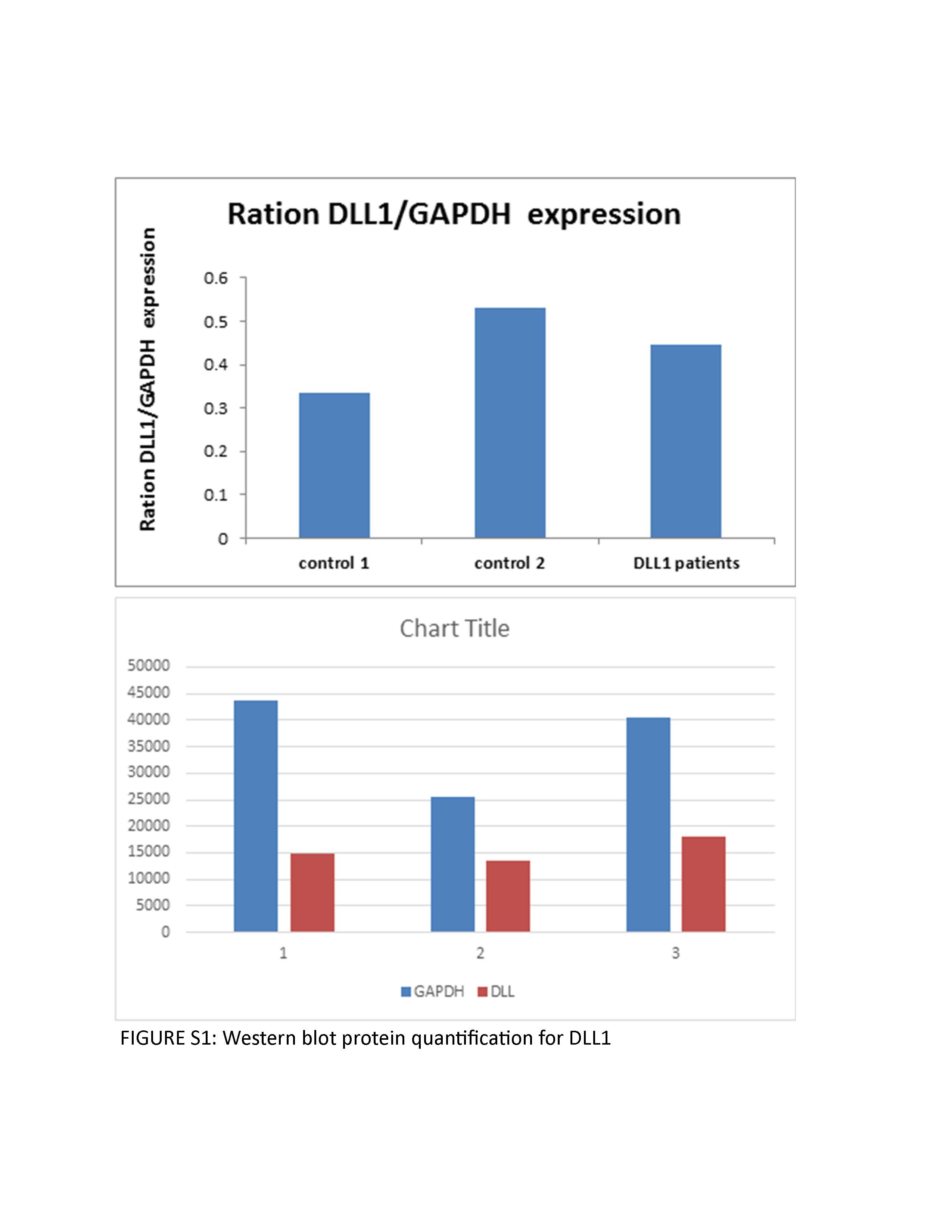

Supplement: Supplementary file 1 [file Image_1.tif]

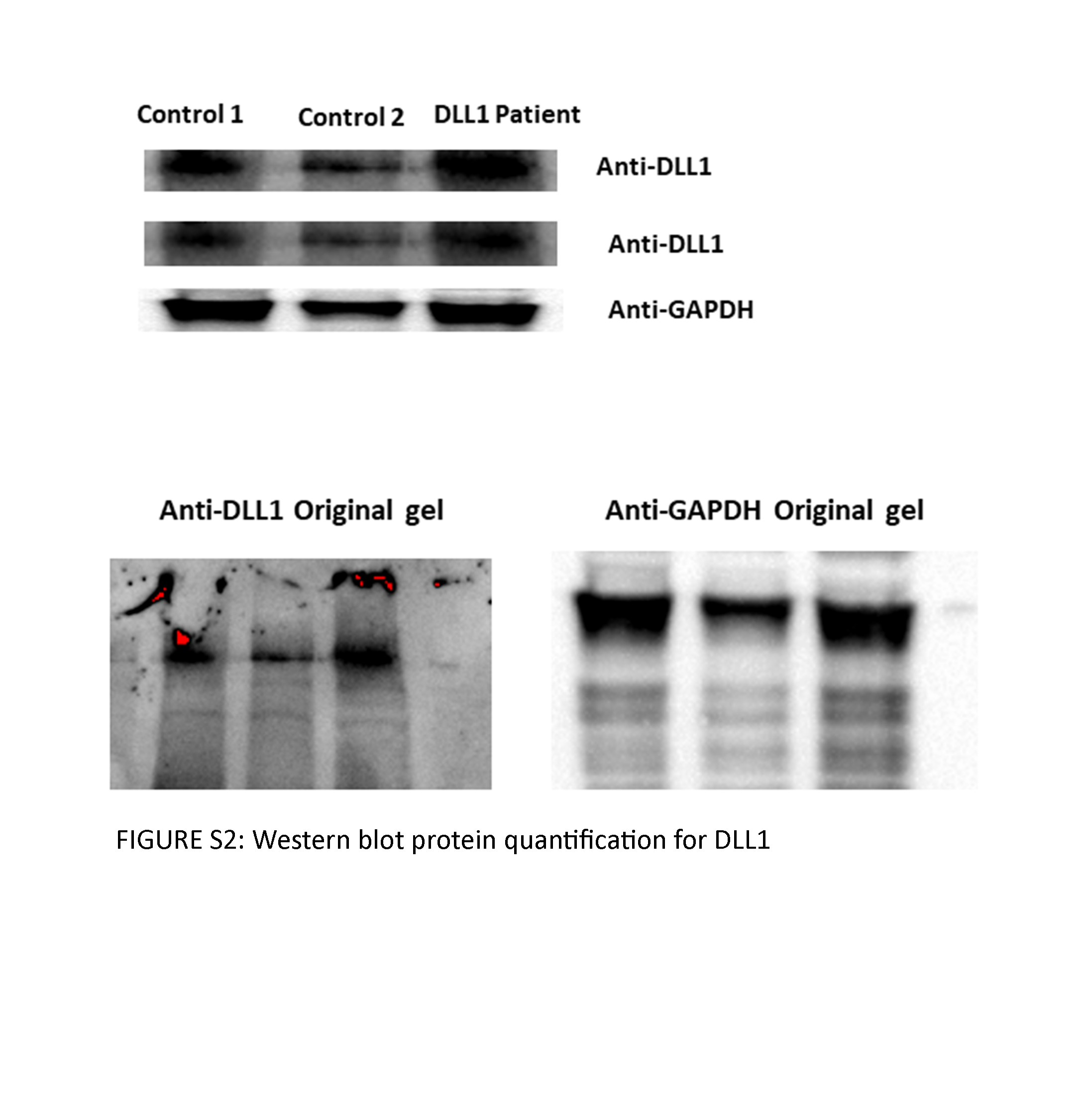

Supplement: Supplementary file 2 [file Image_2.tif]

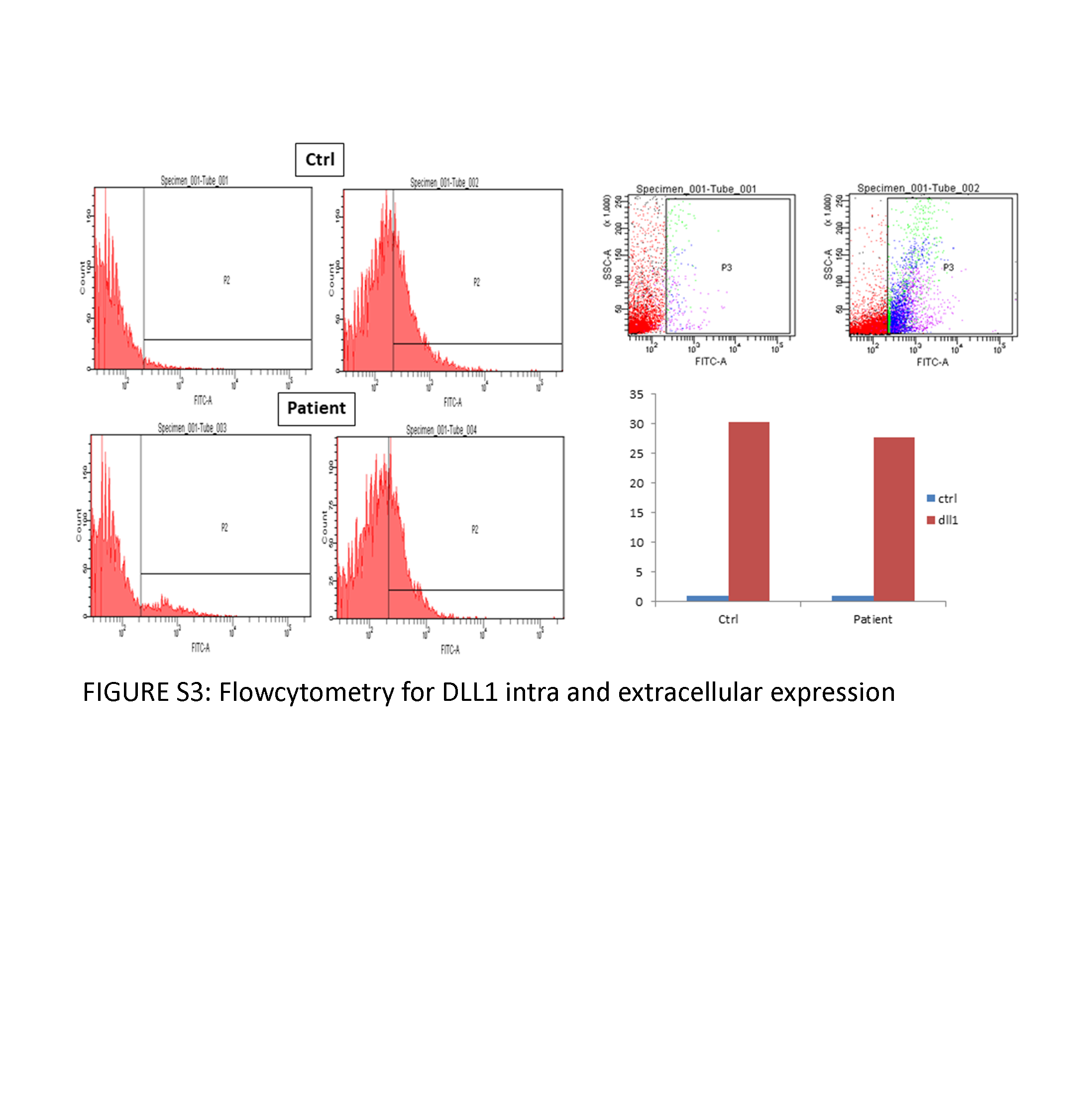

Supplement: Supplementary file 3 [file Image_3.tif]

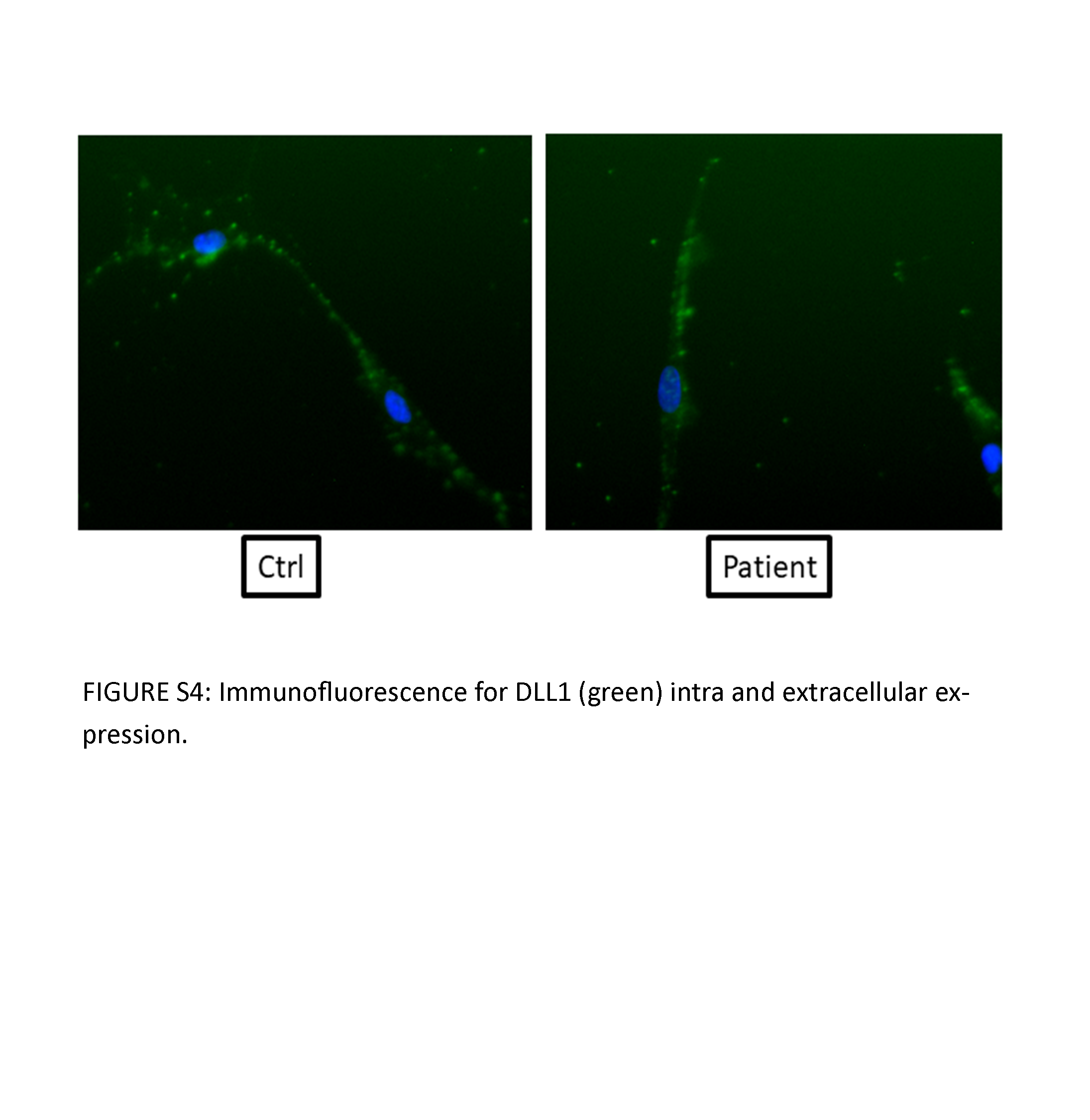

Supplement: Supplementary file 4 [file Image_4.tif]
