# Supplementary material for: Delta Like-1 Gene Mutation: A Novel Cause of Congenital Vertebral Malformation
Source: Front Genet. 2019 Jun 5;10:534. doi: 10.3389/fgene.2019.00534 (PMC6593294; doi:10.3389/fgene.2019.00534)
Supplement: Supplementary file 5 [file Table_1.docx]

| Chromosome | Position | Reference | Altered | Gene | Impact |
| --- | --- | --- | --- | --- | --- |
| 1 | 38167361 | A | AC | CDCA8 | intron_variant |
| 1 | 43023713 | G | GA | CCDC30 | intron_variant&NMD_transcript_variant |
| 10 | 118351098 | A | ATAC | PNLIPRP1 | intron_variant&non_coding_transcript_variant |
| 2 | 188293619 | CA | C | CALCRL | intron_variant |
| 3 | 134085070 | GAAGT | G | AMOTL2 | downstream_gene_variant |
| 5 | 55753822 | C | CA | AC008391.1 | upstream_gene_variant |
| 6 | 167753740 | G | A | TTLL2 | downstream_gene_variant |
| 6 | 168264407 | A | G | MLLT4 | intron_variant&non_coding_transcript_variant |
| 6 | 170592833 | C | T | RP5-894D12.3 | downstream_gene_variant |
| 7 | 75045496 | AG | A | POM121C | downstream_gene_variant |
| X | 117796630 | G | GT | DOCK11 | intron_variant |

Table S1: All identified variants in the whole exome sequencing other than the DLL1:
